# Roles of gas molecules and related factors in knee cartilage injury and repair: a bibliometric visualization analysis of research hotspots

**DOI:** 10.4103/mgr.MEDGASRES-D-26-00163

**Published:** 2026-07-23

**Authors:** Dehua Liu, Yiwen Liu, Xue Wang, Xu Dong, Li Zhang, Gang Ji, Guobin Liu

**Affiliations:** 1 Department of Orthopedics, The First Hospital of Hebei Medical University, Shijiazhuang, Hebei Province, China; 2 Tianjin Key Laboratory of Bone Implant Interface Functionalization and Personality Research, Just Medical Equipment (Tianjin) Co., Ltd., Tianjin, China

**Keywords:** apoptosis, bibliometrics, cartilage injury, chondrocytes, gas molecules, knee joint, medical gases, nitric oxide, oxidative stress, oxygen, reactive oxygen species, repair, visualization analysis

## Abstract

After knee cartilage injury, the self-repair capability is limited, making it prone to progression into osteoarthritis. Recently, gas signaling molecules such as nitric oxide and oxygen, along with reactive oxygen species and other factors associated with oxygen metabolism, have increasingly demonstrated critical roles in the field of knee cartilage injury and repair. However, there are few reports on the evolving research hotspots in this field. Based on the Web of Science Core Collection database, this study conducted a bibliometric and visualization analysis of 245 publications related to the roles of gas molecules and other factors associated with knee cartilage injury and repair. The results reveal a clear evolutionary trend in the research hotspots of this field, characterized by a transition from basic mechanism exploration to translational application transformation. (1) Basic mechanism exploration stage: The keyword co-occurrence network identified a tightly-knit cluster comprising core nodes such as “nitric oxide,” “chondrocytes,” “apoptosis,” “reactive oxygen species,” “oxidative stress,” and “hypoxia.” Co-citation analysis revealed that classic studies had demonstrated that nitric oxide mediates chondrocyte injury and matrix degradation through the inducible nitric oxide synthase–nitric oxide–apoptosis signaling pathway. Subsequent research further clarified the dual regulatory roles of oxygen and reactive oxygen species, confirming the significance of the hypoxia-inducible factor pathway in cartilage metabolism. Burst detection revealed that core literature from this period, such as studies on inducible nitric oxide synthase inhibition and hypoxic culture, maintained a high citation rate for 5–8 years, thereby forming a long-term, stable knowledge foundation in the field. (2) Translational application transformation stage: Emerging translational and biomaterial-related nodes, including “graphene oxide,” “cartilage tissue engineering,” and “nanomedicine,” together with gas-based interventions such as “ozone therapy,” have surged and formed strong interdisciplinary connections with “osteoarthritis” and “cartilage repair.” This shift illustrates a change in perspective from “injury mechanisms” to “repair interventions.” Co-citation analysis confirmed that the most significant recent literature focuses on the relationship between reactive oxygen species, cartilage aging, and osteoarthritis, as well as on nanoscale drug delivery systems for directly clearing nitric oxide and reactive oxygen species. Burst detection revealed that “synergistic intervention of materials and gases” has emerged as the most active frontier hotspot. These findings suggest that gas signaling molecules and oxygen metabolism–related factors are core mediators regulating knee cartilage injury and repair. Research hotspots have shifted from exploring the inducible nitric oxide synthase–nitric oxide–apoptosis signaling pathway and oxidative stress in cartilage injury to translational applications in nanomedicine and biomaterials for cartilage repair. Future research may benefit from elucidating the mechanisms underlying multi-factor interactive networks and promoting the clinical translation of gas-targeted regulatory strategies.

## Graphical abstract

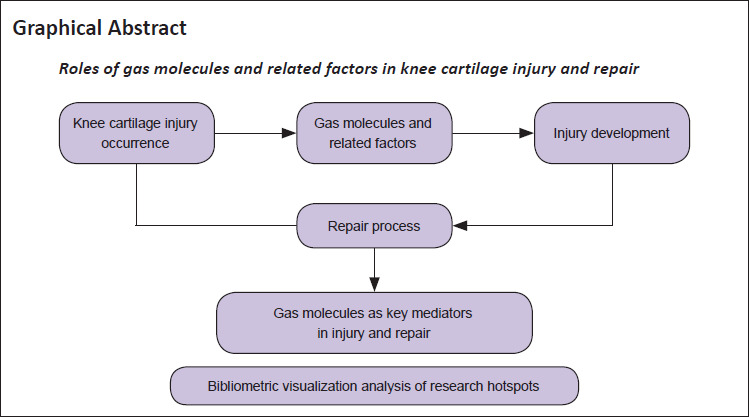

## Introduction

Knee cartilage injury is a common condition in sports medicine and orthopedics.^1^,^2^ The self-repair capacity of injured articular cartilage is very limited, owing to its avascular and aneural nature, as well as the poor proliferative ability of chondrocytes. Consequently, without timely and standardized intervention, such injuries can easily progress to osteoarthritis, resulting in a substantial social and healthcare burden.^2^,^3^,^4^ Notably, recent years have witnessed accumulating evidence confirming the critical role of gas molecules and related regulatory factors in cartilage repair, establishing them as a key research hotspot in this field.

Substantial progress has been made in understanding the basic mechanisms of knee cartilage injury and repair. Endogenous gasotransmitters, such as nitric oxide (NO), oxygen (O_2_), hydrogen (H_2_), and hydrogen sulfide (H_2_S), have been shown to regulate cartilage repair via multiple signaling pathways.^5^,^6^ Among these, H_2_ exerts chondroprotective effects by acting as a selective antioxidant, suppressing the inflammatory microenvironment, and restoring mitochondrial function in chondrocytes.^7^ H_2_S mitigates cartilage degeneration by downregulating matrix metalloproteinases (MMPs) and thereby reducing type II collagen degradation. The development of targeted delivery systems for these molecules has accordingly become a prominent research direction.^8^ At the level of related factors, reactive oxygen species (ROS) have gained increasing recognition for their dual roles in cartilage injury and repair.^9^,^10^ Pro-inflammatory cytokines such as interleukin-1β (IL-1β) and tumor necrosis factor-alpha (TNF-α) are established drivers of cartilage matrix degradation and chondrocyte apoptosis.^11^,^12^ Conversely, transforming growth factor-beta (TGF-β), bone morphogenetic proteins (BMPs), and growth differentiation factor 15 (GDF-15) promote chondrocyte proliferation, differentiation, and matrix synthesis, thereby representing key targets for cartilage regeneration.^13^,^14^,^15^ In parallel, bibliometric and visualization analysis methods have been widely used in medical research to identify emerging trends. Both approaches facilitate quantitative, panoramic assessments of research landscapes, scholarly collaboration networks, and the evolution of thematic foci.^16^,^17^

Significant gaps remain in current research on gas molecules and related factors in knee cartilage injury repair. Specifically, differences in research focus among various molecules, the extent of attention to cross-regulatory networks, and the status of translational research bottlenecks have not been sufficiently quantified or analyzed.^18^,^19^ It should be noted that the literature identified in this study predominantly addresses the degenerative osteoarthritis process, whereas research specifically focused on acute traumatic cartilage injury remains comparatively scarce. To address these issues, this study uses bibliometric and visualization methods to systematically map the global research status, evolving hotspots, and knowledge structure in this field. By identifying core authors, key literature, research frontiers, and emerging topics, this study aims to provide researchers with a clear understanding of the landscape of the field, outline promising future directions, and establish a theoretical foundation for developing gas signaling–based cartilage repair strategies. However, bibliometric evidence for H_2_ and H_2_S remains limited, and this study focuses on molecules with sufficient literature volume for clustering analysis.

## Methods

### Literature search strategy

**Figure 1:**
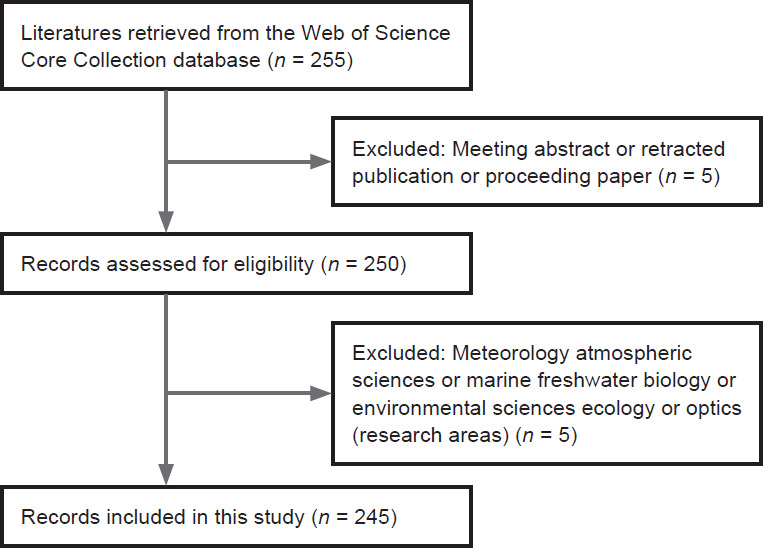
Literature screening flowchart.

A systematic literature search was conducted using the Web of Science Core Collection database on April 1, 2026, with no restrictions on publication date. Inclusion criteria: studies on the roles of gas molecules and related factors in knee joint cartilage injury and repair. Included document types were original articles, review articles, and early access publications. Meeting abstracts, retracted publications, and proceeding papers were excluded. Additionally, records from the research areas of meteorology atmospheric sciences, marine freshwater biology, environmental sciences ecology, and optics were excluded. A total of 245 articles, including preprints and early access publications from 2025 to 2026, were retrieved. The detailed search strategy is presented in **Table 1**, and the document screening process is shown in **Figure 1**.

**Table 1:** Literature search strategy for the Web of Science Core Collection database

#	Search query	Results
1	TI=(gas OR gases OR Oxygen* OR Hypoxia* OR “Nitric Oxide*” OR “Carbon Dioxide*” OR “Hyperbaric Oxygen*” OR Ozone* OR “Hydro* Peroxide*” OR “Nitrous Oxide*” OR “Carbon Monoxide*” OR “Sulfur Dioxide*” OR “Nitrogen Dioxide*” OR Hyperoxia* OR Argon* OR Helium* OR “Compressed Air*” OR “Pressurized Air*” OR Hydrogen* OR Oxide* OR Hydride* OR Nitrogen* OR Gasotransmitter* OR Gaseous* OR “Aero-ionotherapy” OR “Air ioniz*” OR “Ioniz* Air*”)	1706405
2	TS=(“Chondro* Implantation” OR “Chondrogenesis” OR “Cartilage Regeneration” OR “Cartilage repair” OR “Chondral reconstruction” OR “Cartilage recombination” OR “Chondroplasty” OR “Osteochondral Repair” OR “Microfracture” OR “Cartilage” OR “Chondrocyte” OR “Chondroblast” OR “Cartilage tissue engineering” OR “Meniscus”)	144061
3	TS=(“Knee Joint*” OR Knee* OR “Tibiofemoral Joint” OR “Femorotibial Joint” OR “Articulatio Genus”)	225032
4	#3 AND #2 AND #1	255
5	#3 AND #2 AND #1 and Meeting Abstract or Retracted Publication or Proceeding Paper (Exclude – Document Types)	250
6	#3 AND #2 AND #1 and Meeting Abstract or Retracted Publication or Proceeding Paper (Exclude – Document Types) and Meteorology Atmospheric Sciences or Marine Freshwater Biology or Environmental Sciences Ecology or Optics (Exclude – Research Areas)	245

### Data collection and processing

The bibliographic information of all 245 articles, including titles, authors, affiliations, countries, journals, keywords, references, and other relevant fields, was exported in plain text format as “download_1-245.txt.” Subsequently, synonym replacement was applied (e.g., “ROS” was replaced with “reactive oxygen species”), and keywords were standardized by converting plural forms to singular (e.g., “chondrocytes” to “chondrocyte”). The standardized data were then saved as a new text document for subsequent analyses. We manually verified that the excluded research areas contained no relevant records.

### Use of visualization tools and data analysis

The standardized txt document was imported into VOSviewer (v1.6.20) and CiteSpace (v6.4.R2) to conduct bibliometric and visualization analyses, covering publication volume, contributing countries, research institutions, core journals, keyword co-occurrence patterns, and citation burst detection.

VOSviewer 1.6.20 was primarily designed for constructing and visualizing collaboration and co-occurrence networks.^20^ Its collaboration network analysis function evaluates research collaboration strength among countries and institutions, where node size indicates publication output or collaboration frequency, line thickness reflects collaboration intensity, and node colors differentiate distinct clusters.^21^,^22^,^23^ Its co-occurrence analysis function extracts keywords from the literature to build a co-occurrence network, with node size representing keyword frequency and lines indicating co-occurrence, thereby revealing research hotspots and their structural patterns.^24^,^25^,^26^ The resulting keyword visualization network is exported as a “xxx.net” file and further optimized using Gephi 0.9.2 software.^27^ In our analysis, we exported the VOSviewer network file to Gephi 0.9.2 and applied the ForceAtlas2 layout algorithm for spatial optimization, followed by modularity class calculation. Additionally, the density view provides a visual representation of the distribution density of network elements (e.g., institutions, keywords), facilitating the identification of core research areas.^28^

CiteSpace 6.4.R2 focuses on analyzing dynamic evolution, burst detection, and knowledge bases.^29^,^30^ Its core functions include journal dual-map overlay analysis, which visualizes interdisciplinary knowledge flow by mapping relationships between citing and cited journals.^31^,^32^ Co-citation analysis constructs co-citation networks and clustering maps to identify the knowledge base, core literature, and research frontiers.^33^,^34^ The co-citation cluster maps and timeline visualizations presented in this study were generated using this approach. Burst detection identifies keywords, references, or authors with a sudden surge in citation or co-occurrence frequency, thereby revealing emerging research hotspots and their evolution.^35^,^36^ Timeline visualization expands clustering results along a timeline to illustrate the developmental trajectories and interconnections of various research themes.^37^,^38^,^39^ For clustering quality assessment, CiteSpace provides two key parameters: the modularity *Q* value (where *Q* > 0.3 indicates a meaningful cluster division) and the weighted mean silhouette *S* value (where *S* > 0.5 reflects high cluster homogeneity and thematic consistency).^40^,^41^

## Results

### Publication volume

**Figure 2:**
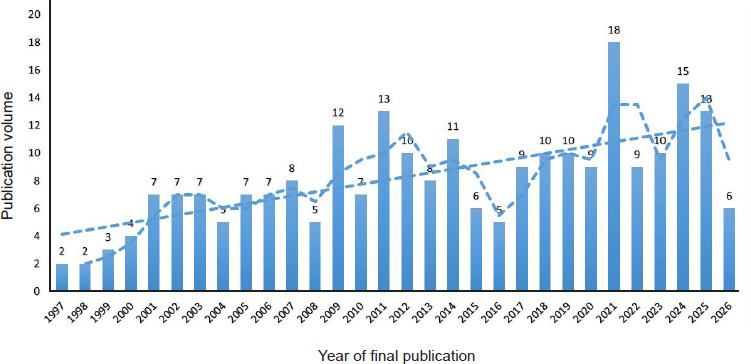
Annual publication trend of research on the roles of gas molecules and related factors in knee cartilage injury and repair.

The overall publication volume in this field shows an upward trend, with the highest number of publications recorded in 2021 at 18 articles, followed by 15 articles in 2024 and 13 articles in 2025 (**Figure 2**). Data from 2025–2026 include early-access publications and may not yet reflect full citation impact.

### Contributing countries

**Figure 3:**
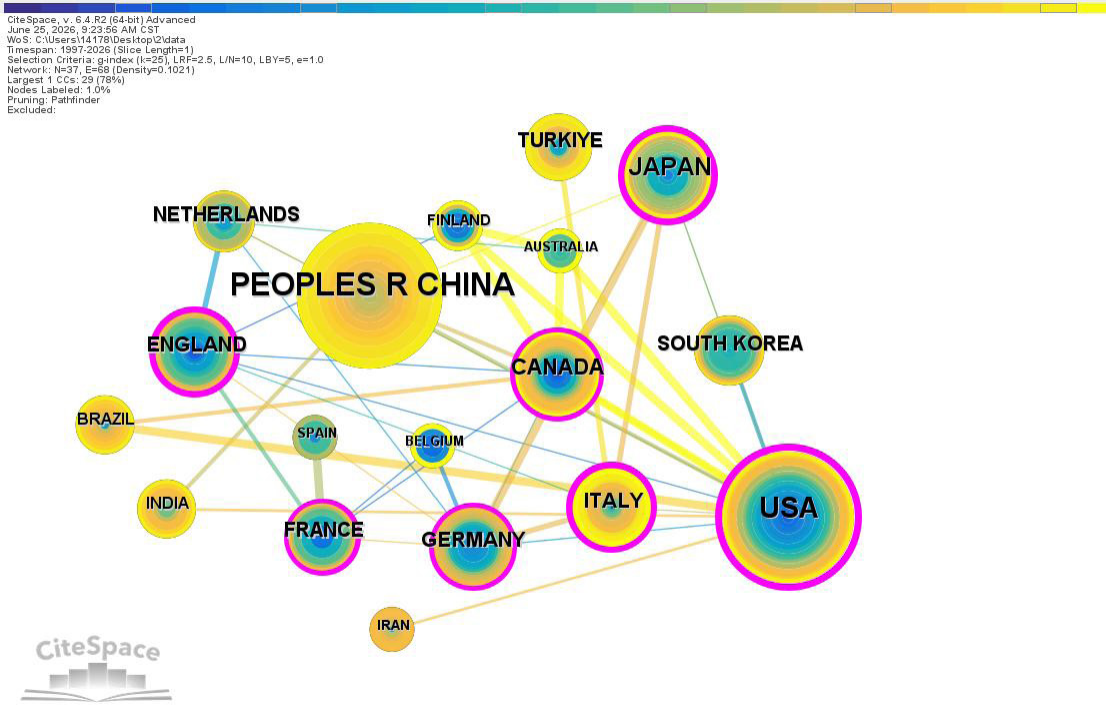
Country analysis of research on gas molecules and related factors in knee cartilage injury and repair. Visualization network diagram of publication volume by country. The area/diameter of the circular nodes in the diagram corresponds to the publication count for each country/region. Larger nodes indicate a greater number of academic papers published in this research field, reflecting a larger scale of scientific output. The node rings (annual rings) consist of colored concentric circles representing the distribution of publication volume over different years for each country. Thicker rings correspond to higher publication volumes for the respective years. Thicker connecting lines indicate stronger collaborative publication relationships and more co-authored papers between countries. The color of the connecting lines corresponds to the timing of collaborations, with warmer colors (yellow/orange) representing more recent collaborations and cooler colors (blue/purple) indicating earlier collaborations.

As shown in **Figure 3** and **Table 2**, China is the leading country in terms of publication volume in this field, contributing a total of 71 articles. The United States has the highest total citation count (2742 citations), indicating the strongest academic influence. China ranks second in academic influence, demonstrating strong development potential. Regarding international collaboration, the United States (centrality = 0.40), England (0.37), Italy (0.35), and Germany (0.31) exhibit relatively high centrality, with the United States serving as the core hub. European and North American countries dominate the field, having initiated research earlier and established themselves as pioneers. Despite ranking high in both publication volume and citation counts, China has a centrality of only 0.08, indicating that its role as an international bridge remains weaker than that of traditional powerhouses in Europe and North America. Since 2006, Asian countries, such as China and South Korea, have rapidly emerged as significant contributors in the later stages of development in this field.

**Table 2:** Comparative analysis of publication indicators by country for research on gas molecules and related factors in knee cartilage injury and repair

Country	Count	Citation	Centrality	Year
PEOPLES R CHINA	71	1809	0.08	2006
USA	47	2742	0.40	1997
JAPAN	25	823	0.16	2000
CANADA	15	835	0.16	1999
GERMANY	14	1096	0.31	2003
ENGLAND	14	968	0.37	1997
ITALY	14	463	0.35	1998
SOUTH KOREA	12	361	0.00	2006
TURKEY	12	143	0.00	2003
FRANCE	11	548	0.08	1999
NETHERLANDS	11	300	0.09	2000
BRAZIL	7	136	0.00	2006
INDIA	7	100	0.00	2014
FINLAND	6	121	0.09	2001
IRAN	5	50	0.00	2007

### Research institutes

As shown in **Table 3**, Scripps Research Institute, Sichuan University, University of Alberta, and University of California, San Diego are the leading institutions in this field, each with six publications, making them high-output organizations. Although Sichuan University and University of Alberta rank highest in publication volume, their total citation counts are only 28 and 27, respectively, which are significantly lower than those of Scripps Research Institute (119 citations) and University of California, San Diego (80 citations). This indicates that some high-output institutions, despite their productivity, have relatively low academic impact.

**Table 3:** Comparative analysis of publication indicators by institute for research on the roles of gas molecules and related factors in knee cartilage injury and repair

Institute	Publication	Citation	Total link strength
Scripps Research Institute	6	119	9
Sichuan University	6	28	0
University of Alberta	6	27	0
University of California, San Diego	6	80	8
Duke University	4	328	0
Kyoto Prefectural University of Medicine	4	193	4
Tampere University Hospital	4	96	4
University of Kiel	4	44	2
University of Pittsburgh	4	60	1
Centre Hospitalier de l'Université de Montréal	3	164	0
Hallym University	3	75	1
Harvard University	3	84	1
Hebei University of Technology	3	42	5
Peking University	3	92	1
Seoul National University	3	671	0
Tianjin University of Traditional Chinese Medicine	3	29	5
University of Erlangen-Nuremberg	3	9	3
University of Manchester	3	473	0
McGill University	3	295	0
University of Queensland	3	154	3
University of São Paulo	3	3	1
University of Tampere	3	66	4

Seoul National University ranks first in total citations, with 671, followed by University of Manchester (473), Duke University (328), and McGill University (295). Although these institutions have relatively fewer publications, they serve as academic benchmarks due to their high-impact research. However, institutions with high citation counts generally exhibit low collaboration activity. For example, Seoul National University, University of Manchester, and Duke University each have a total link strength of 0, suggesting a focus on in-depth research within their own institutions and very limited cross-institutional collaboration.

**Figure 4:**
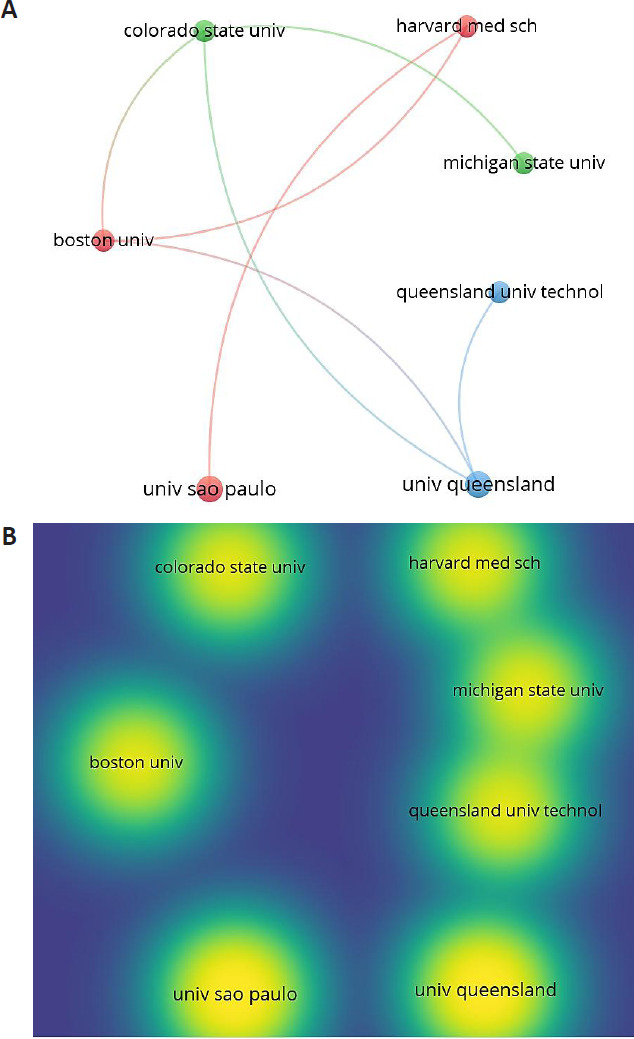
Institutional collaboration network diagram of the research on the roles of gas molecules and related factors in knee cartilage injury and repair. (A) Visualization of the institutional collaboration network. Nodes represent research institutions, and lines indicate collaboration relationships. Different colors distinguish three independent collaboration clusters, providing a clear representation of the collaboration patterns among institutions. (B) Visualization of institutional collaboration density. A yellow-to-blue gradient displays the strength of collaborations, confirming the barriers between clusters. This diagram clearly shows the collaboration activity levels and network distribution among the institutions.

There have been relatively few collaborative publications among institutions; the main collaborating institutions include Boston University, Colorado State University, Harvard Medical School, Michigan State University, University of Queensland, and University of São Paulo, as shown in **Figure 4**.

### Journals

**Figure 5:**
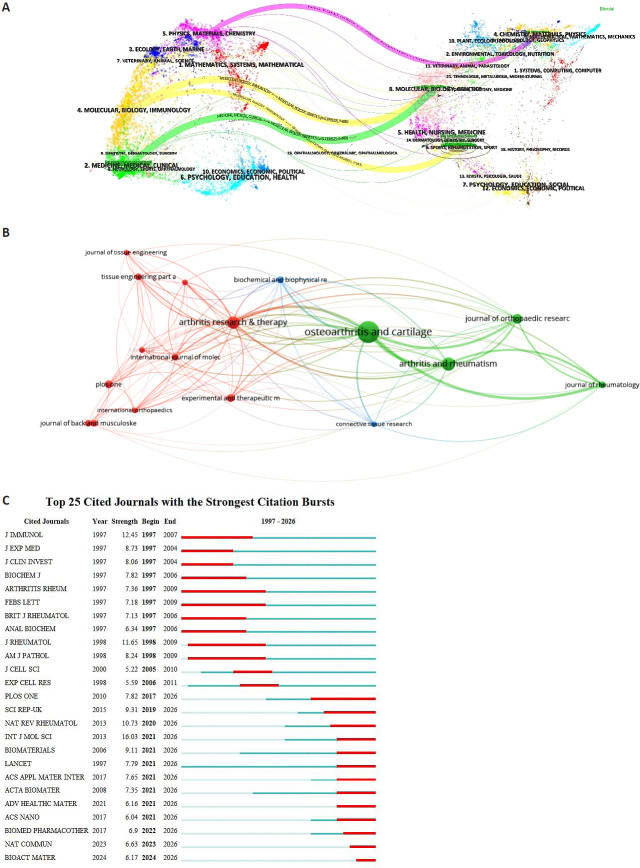
Comparative analysis of the research on the roles of gas molecules and related factors in knee cartilage injury and repair by journal. (A) Dual-map overlay: The left side represents the citing side, which displays the discipline clusters of the current research publications, with the size of the point clusters corresponding to the volume of publications in each discipline, and colors distinguishing different discipline clusters. The right side represents the cited side, which shows the discipline clusters of the references cited by these citing publications, forming the knowledge foundation of the field. The colorful connecting lines in the middle indicate the direction of citations, with line thickness representing citation intensity and colors matching the corresponding disciplines on the left, visually demonstrating the knowledge transfer relationships between disciplines. (B) Journal network visualization: The larger the node, the higher the publication volume of the journal. The thicker the lines, the closer the citation relationships between journals. Different colors represent different journal clusters. (C) Journal co-citation burst diagram: The light blue bands represent the complete time range, while the red bands indicate the citation burst periods of the journals.

**Figure 5A** shows that the clusters in the fields of medicine, biology, and mathematics are currently the main areas of scientific publications. The biomedical cluster is the most highly cited and serves as the core foundation for knowledge production.

As shown in **Figure 5B** and **Table 4**, *Osteoarthritis and Cartilage* is the absolute core journal in this field, with 23 publications, 1690 total citations, and a total link strength of 36, making it the largest node in the network. It not only leads in academic impact but also serves as a hub in the journal association network, establishing itself as a top authority in the study of osteoarthritis and cartilage. Following closely is *Arthritis and Rheumatism*, with 11 publications, 1484 citations, and a total link strength of 35, making it the second core journal and possessing significant academic impact in the field of rheumatic arthritis.

**Table 4:** Comparative analysis of research on the roles of gas molecules and related factors in knee cartilage injury and repair by journal

Journal	Publication	Citation	Total link strength
*Osteoarthritis and Cartilage*	23	1690	36
*Arthritis and Rheumatism*	11	1484	35
*Arthritis Research Therapy*	10	655	19
*Journal of Orthopaedic Research*	7	315	10
*Experimental and Therapeutic Medicine*	5	128	8
*PLoS One*	5	219	1
*Biochemical and Biophysical Research Communications*	4	77	7
*International Journal of Molecular Sciences*	4	31	4
*Journal of Back and Musculoskeletal Rehabilitation*	4	63	0
*Journal of Orthopaedic Surgery and Research*	4	36	0
*Journal of Rheumatology*	4	391	13
*Tissue Engineering Part A*	4	92	4
*BMC Musculoskeletal Disorders*	3	56	6
*Cartilage*	3	76	2
*Connective Tissue Research*	3	46	3
*International Orthopaedics*	3	107	4
*Journal of Tissue Engineering and Regenerative Medicine*	3	49	4

**Figure 5C** shows a co-citation burst diagram of journals, highlighting the evolution of research hotspots over the past 30 years. The red burst intervals reveal two distinct phases: (1) Traditional classical period (1997–2011): Dominated by traditional disciplines such as basic medicine, immunology, and rheumatology, this phase establishes the foundation for the field. Representative journals (*Journal of Immunology*, *Journal of Experimental Medicine*, *Arthritis and Rheumatism*) showed high burst intensity (e.g., *Journal of Immunology*, intensity 12.45) and long burst durations (8–10 years), indicating the sustained importance of traditional basic disciplines. (2) Emerging interdisciplinary period (2017–2026): This phase marks a clear shift toward biomaterials, nanomedicine, molecular science, and open-access journals as core hotspots, reflecting an interdisciplinary expansion. Key journals include *International Journal of Molecular Sciences* (burst intensity 16.03, the highest in the diagram), *Biomaterials*, *ACS Nano*, and *PLoS One*. Bursts are concentrated in 2021–2026, with shorter durations (typically < 5 years) and faster hotspot turnover. While established journals such as *The Lancet* maintain their influence, numerous new interdisciplinary journals at the materials–medicine interface have emerged, indicating a transition from “basic mechanisms” to “translational applications and materials innovation.”

### Keywords

**Table 5** presents the main key words and their frequencies in this research field. The statistical data in **Table 5** reflect the core hotspots and contexts of research related to osteoarthritis. “Osteoarthritis” appears 83 times with a total link strength of 137, making it the absolute core theme of the entire research area. “Chondrocyte,” “nitric oxide,” and “cartilage” also rank high in both frequency and link strength, indicating that research focuses on chondrocytes, nitric oxide, and cartilage tissue. The frequent occurrence of “reactive oxygen species,” “oxidative stress,” “apoptosis,” and “hypoxia” suggests that oxidative stress, hypoxia, and cell apoptosis are key research directions in the pathogenesis of osteoarthritis. The research is primarily focused on knee osteoarthritis, while also addressing pathological structures or related factors such as inflammation, cytokines, the meniscus, and collagen. Furthermore, terms such as “ozone/ozone therapy” and “graphene oxide” reflect emerging trends in innovative materials and physical therapeutic methods for cartilage repair.

**Table 5:** Keyword co-occurrence frequency in research on the roles of gas molecules and related factors in knee cartilage injury and repair

Keyword	Occurrence	Total link strength
Osteoarthritis	83	137
Chondrocyte	38	84
Nitric oxide	35	78
Cartilage	23	45
Reactive oxygen species	18	27
Apoptosis	16	39
Hypoxia	16	34
Oxidative stress	12	27
Articular cartilage	11	20
Knee	10	17
Knee osteoarthritis	10	15
Inflammation	9	22
Meniscus	8	22
Collagen	7	17
Graphene oxide	7	7
Arthritis	6	14
Ozone	6	14
Cartilage repair	5	6
Cytokines	5	13
Extracellular matrix	5	10
Oxygen tension	5	12
Ozone therapy	5	12

“Occurrence” refers to the frequency of keyword appearances, while “Total link strength” indicates the total number of co-occurrences of the keyword with other keywords (including repeated co-occurrences).

**Figure 6:**
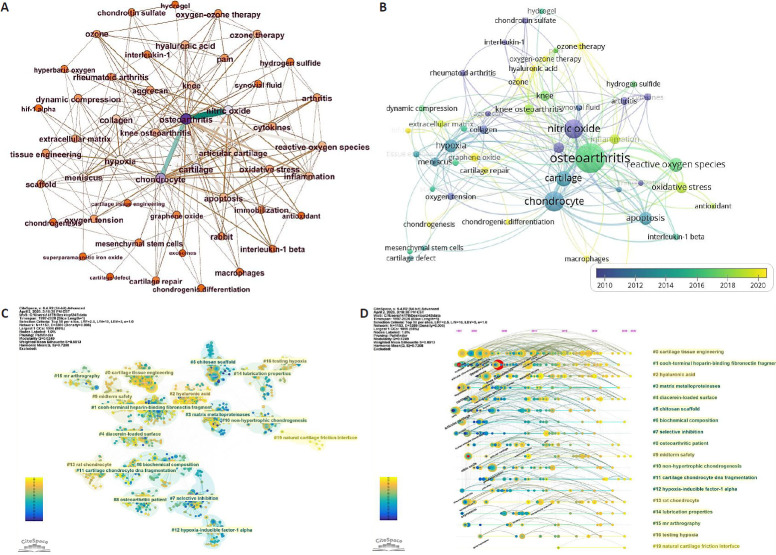
Visualization analysis of keywords related to the roles of gas molecules and related factors in knee cartilage injury and repair. (A) Keyword co-occurrence visualization network. Different colors represent distinct thematic clusters. (B) Keyword overlay visualization network. Different colors represent the average publication year. In A and B, each labeled circular node represents a keyword, with larger nodes indicating higher keyword frequency and thicker lines representing stronger connections between keywords. (C) Keyword clustering visualization map. Irregularly shaped colored blocks represent keyword clusters. Each cluster label represents the core thematic direction of that keyword cluster. Each node represents a keyword, with node size corresponding to keyword occurrence or frequency. Lines between nodes indicate keyword co-occurrence relationships, with thicker lines representing stronger co-occurrence strength. (D) Keyword clustering timeline. The timeline map shows the temporal evolution of keyword clusters from 1997 to 2026. Each node represents a keyword, and node size reflects keyword occurrence or frequency. The horizontal axis represents time, and different horizontal lines represent distinct keyword clusters. The colors of nodes and links indicate the time period in which the keywords appeared or were most active. Links between nodes indicate co-occurrence relationships among keywords.

As shown in **Figure 6A**, “osteoarthritis” occupies the central position, surrounded by two major pathological cores “cartilage” and “chondrocytes.” This framework encompasses three primary research dimensions: pathogenesis, therapeutic interventions, and tissue repair, thereby clearly outlining the core structure of osteoarthritis research. The research hotspots can be classified into three levels: (1) Pathological mechanisms: Key areas of focus include inflammation, oxidative stress, apoptosis, cytokines (e.g., interleukins), and reactive oxygen species. These factors highlight the core pathological drivers of osteoarthritis. (2) Therapeutic interventions: Clinical research hotspots involve ozone/ozone therapy, hyaluronic acid, chondroitin sulfate, and antioxidants. Moreover, emerging approaches such as hyperbaric oxygen therapy and mesenchymal stem cells are attracting considerable attention. (3) Tissue engineering and repair: Core regenerative medicine topics include cartilage repair, chondrogenic differentiation, scaffolds, and tissue engineering. These are cutting-edge therapeutic strategies for osteoarthritis, bridging multiple disciplines such as clinical medicine, materials science (e.g., graphene oxide and iron oxide nanoparticles), cell biology, and immunology, thereby indicating the interdisciplinary nature of osteoarthritis research. As shown in **Figure 6B**, keyword hotspots during the 2010–2015 period were mainly concentrated on basic pathological topics, such as hypoxia, the meniscus, and cartilage repair. In contrast, during the 2016–2020 period, keyword hotspots shifted toward oxidative stress, antioxidants, ozone therapy, and stem cell regeneration. This evolution reflects a transition in research focus from mechanistic studies toward clinical interventions and regenerative medicine.

As shown in **Figure 6C**, the keyword cooccurrence network consists of the 18 largest clusters, which are as follows: Cluster #0: cartilage tissue engineering; Cluster #1: COOH-terminal heparin-binding fibronectin fragment; Cluster #2: hyaluronic acid; Cluster #3: matrix metalloproteinases; Cluster #4: diacerein-loaded surface; Cluster #5: chitosan scaffold; Cluster #6: biochemical composition; Cluster #7: selective inhibition; Cluster #8: osteoarthritic patient; Cluster #9: midterm safety; Cluster #10: non-hypertrophic chondrogenesis; Cluster #11: cartilage chondrocyte DNA fragmentation; Cluster #12: hypoxia-inducible factor-1 alpha; Cluster #13: rat chondrocyte; Cluster #14: lubrication properties; Cluster #15: MR arthrography; Cluster #16: testing hypoxia; Cluster #19: natural cartilage friction interface. The top-ranked keyword by occurrence is “cartilage” (Cluster #3), with 47 citations, followed by “knee osteoarthritis” (Cluster #1) with 45 citations and “articular cartilage” (Cluster #5) with 44 citations. The fourth is “expression” (Cluster #13) with 42 citations, the fifth is “knee” (Cluster #0) with 41 citations, and the sixth is “nitric oxide” (Cluster #9) with 39 citations. The seventh is “osteoarthritis” (Cluster #0) with 31 citations, the eighth is “chondrocyte” (Cluster #2) with 25 citations, the ninth is “gene-expression” (Cluster #10) with 22 citations, and the tenth is “oxidative stress” (Cluster #1) with 22 citations.

**Figure 6C** shows that Cluster #0 focuses on cartilage tissue engineering as the main research theme. Clusters #3 (matrix metalloproteinases), #12 (hypoxia-inducible factor-1 alpha), #16 (testing hypoxia), and #10 (non-hypertrophic chondrogenesis) concentrate on core biological issues such as the regulation of chondrocyte differentiation, responses to the microenvironment (hypoxia), and mechanisms of inflammatory injury. These clusters form the theoretical foundation of the field, with hypoxia-related research gaining significant momentum in recent years and becoming a key regulatory target for cartilage regeneration. The theme of innovations in biomaterials and intervention technologies includes Clusters #2 (hyaluronic acid), #5 (chitosan scaffold), #4 (diacerein-loaded surface), #19 (natural cartilage friction interface), and #14 (lubrication properties). These represent the core directions in material development, ranging from traditional scaffold materials to functional drug delivery surfaces, and from biomimetic artificial materials to natural cartilage interface mechanisms. Additionally, the lubrication mechanisms of cartilage have emerged as a new interdisciplinary hotspot, driving improvements in the performance of joint repair materials.

The theme of clinical translation and application includes Clusters #8 (osteoarthritic patient), #9 (midterm safety), and #15 (MR arthrography), reflecting the trend of research moving toward clinical application. This involves safety assessments and optimization of imaging diagnostics related to osteoarthritis treatment. Meanwhile, studies on targets such as Cluster #1 (COOH-terminal heparin-binding fibronectin fragment) and Cluster #7 (selective inhibition) provide a foundation for the development of targeted therapies, creating a closed loop from basic research to clinical intervention. Future breakthroughs will focus on hypoxic microenvironments, cartilage lubrication, and targeted interventions.

**Figure 6D** presents the development trends of keyword clustering directions in this field from 1997 to 2026. (1) Core technology direction: Cluster #0 (cartilage tissue engineering) is the primary core theme and serves as the foundation of the entire field, linking all downstream sub-research areas. (2) Biomaterials and scaffolds: Clusters #1 (cartilage basement binding fibronectin layer), #6 (cartilage scaffold), and #12 (hydrogel) represent the core material directions. Hydrogels have become a research hotspot in recent years due to their ability to mimic the extracellular matrix and support cell adhesion and proliferation (indicated by yellow/red nodes). Decellularized matrices focus on the biomimetic modification of natural biological materials. (3) Pathology and intervention targets: Cluster #5 (matrix metalloproteinases) plays a critical role as key enzymes in cartilage degeneration and repair. studying the regulatory mechanisms of matrix metalloproteinases is essential for inhibiting cartilage degradation. Clusters #10 (hypertrophic chondrocytes) and #11 (dedifferentiation) focus on maintaining chondrocyte function and understanding degeneration mechanisms, which are central to addressing osteoarthritis. (4) Regeneration strategies: Clusters #13 (chondrocyte inductive factor alpha) and #15 (osteogenic patterning) focus on the regulation of stem cell differentiation into cartilage and bone lineages, which is the core biological mechanism of tissue engineering. Clusters #16 (education properties) and #17 (testing hypoxia) explore the effects of physical signals, such as hypoxia and mechanical stimulation, on cartilage regeneration from the perspective of microenvironmental regulation.

### Literature hotspot analysis

The top 20 highly cited publications were analyzed^42^,^43^,^44^,^45^,^46^,^47^,^48^,^49^,^50^,^51^,^52^,^53^,^54^,^55^,^56^,^57^,^58^,^59^,^60^,^61^ (**Table 6**). The findings indicate that gas molecules, along with related inflammatory and regulatory factors, play complex and critical roles in the pathogenesis of knee cartilage injury and osteoarthritis. Furthermore, they are not only involved in the mechanisms underlying cartilage injury but also offer important therapeutic targets and insights for repair.

**Table 6:** Top 20 highly cited publications on the roles of gas molecules and related factors in knee cartilage injury and osteoarthritis

First author (publication year)	Article title	Source journal	Document type	Times cited, WoS Core
Henrotin (2005)^42^	Oxygen and reactive oxygen species in cartilage degradation: friends or foes?	*Osteoarthritis and Cartilage*	Review	420
Hashimoto (1998)^43^	Chondrocyte apoptosis and nitric oxide production during experimentally induced osteoarthritis	*Arthritis and Rheumatism*	Article	307
Melchiorri (1998)^44^	Enhanced and coordinated in vivo expression of inflammatory cytokines and nitric oxide synthase by chondrocytes from patients with osteoarthritis	*Arthritis and Rheumatism*	Article	215
Clements (2003)^45^	Gene deletion of either interleukin-1β, interleukin-1β-converting enzyme, inducible nitric oxide synthase, or stromelysin 1 accelerates the development of knee osteoarthritis in mice after surgical transection of the medial collateral ligament and partial medial meniscectomy	*Arthritis and Rheumatism*	Article	186
LeGrand (2001)^46^	Interleukin-1, tumor necrosis factor α, and interleukin-17 synergistically up-regulate nitric oxide and prostaglandin E2 production in explants of human osteoarthritic knee menisci	*Arthritis and Rheumatism*	Article	183
Grimshaw (2000)^47^	Bovine articular chondrocyte function *in vitro* depends upon oxygen tension	*Osteoarthritis and Cartilage*	Article	180
Khan (2007)^48^	Hypoxic conditions increase hypoxia-inducible transcription factor 2α and enhance chondrogenesis in stem cells from the infrapatellar fat pad of osteoarthritis patients	*Arthritis Research Therapy*	Article	174
Ryu (2011)^49^	Interleukin-6 plays an essential role in hypoxia-inducible factor 2α-induced experimental osteoarthritic cartilage destruction in mice	*Arthritis and Rheumatism*	Article	163
Attur (1997)^50^	Interleukin-17 up-regulation of nitric oxide production in human osteoarthritis cartilage	*Arthritis and Rheumatism*	Article	158
Pelletier (1999)^51^	Selective inhibition of inducible nitric oxide synthase in experimental osteoarthritis is associated with reduction in tissue levels of catabolic factors	*Journal of Rheumatology*	Article	149
Chan (2005)^52^	Glucosamine and chondroitin sulfate regulate gene expression and synthesis of nitric oxide and prostaglandin E2 in articular cartilage explants	*Osteoarthritis and Cartilage*	Article	138
Jing (2008)^53^	In vivo MR imaging tracking of magnetic iron oxide nanoparticle labeled, engineered, autologous bone marrow mesenchymal stem cells following intra-articular injection	*Joint Bone Spine*	Article	130
Coimbra (2004)^54^	Hypoxia inducible factor-1 alpha expression in human normal and osteoarthritic chondrocytes	*Osteoarthritis and Cartilage*	Article	120
Yan (2023)^55^	Nanomedicines reprogram synovial macrophages by scavenging nitric oxide and silencing CA9 in progressive osteoarthritis	*Advanced Science*	Article	113
Li (2013)^56^	The complex effects of the slow-releasing hydrogen sulfide donor GYY4137 in a model of acute joint inflammation and in human cartilage cells	*Journal of Cellular and Molecular Medicine*	Article	108
Takahashi (2000)^57^	Effect of hyaluronan on chondrocyte apoptosis and nitric oxide production in experimentally induced osteoarthritis	*Journal of Rheumatology*	Article	103
Pelletier (2001)^58^	Chondrocyte death in experimental osteoarthritis is mediated by MEK 1/2 and p38 pathways: Role of cyclooxygenase-2 and inducible nitric oxide synthase	*Journal of Rheumatology*	Article	101
Xue (2024)^59^	Reactive oxygen species (ROS)-mediated M1 macrophage-dependent nanomedicine remodels inflammatory microenvironment for osteoarthritis recession	*Bioactive Materials*	Article	99
Chen (2017)^60^	Graphene oxide/PVA inorganic/organic interpenetrating hydrogels with excellent mechanical properties and biocompatibility	*Carbon*	Article	99
Thoms (2013)^61^	Hypoxia promotes the production and inhibits the destruction of human articular cartilage	*Arthritis and Rheumatism*	Article	99

“Times cited, WoS Core” represents the total citation counts of an article within the Web of Science Core Collection database, reflecting the global academic impact of the publication.

The research presented in **Table 6** reveals that gas molecules such as O_2_, NO, ROS, and H_2_S interact with related factors including IL-1β, TNF-α, HIF-1α, and HIF-2α to jointly influence cartilage homeostasis, injury progression, and repair outcomes. Among these, the review article by Henrotin (cited 420 times)^42^ lays the foundation for understanding the roles of oxygen and ROS in cartilage metabolism and is the most cited article.

O_2_, as a key environmental gas factor, exerts a dual regulatory effect on cartilage metabolism. Grimshaw and Mason^47^ (cited 180 times) demonstrated that physiological hypoxia (5–10% O_2_) can maintain normal chondrocyte function, whereas severe hypoxia (< 0.1% O_2_) significantly impairs cellular function. Henrotin et al.^42^ (cited 420 times) also pointed out that moderate hypoxia is beneficial for maintaining the chondrocyte phenotype.

Hypoxia-inducible factors (HIF-1α and HIF-2α) are core mediators of chondrocyte adaptation to hypoxic environments. Coimbra et al.^54^ (cited 120 times) found that HIF-1α can be induced by hypoxia and TNF-α, mediating anti-catabolic responses. Thoms^61^ (cited 99 times) revealed that HIF-2α can upregulate SOX9 to promote cartilage formation. Ryu et al.^49^ (cited 163 times) discovered that IL-6 regulation can activate the HIF-2α/IL-6/MMPs signaling axis, thereby accelerating cartilage degradation. Khan^48^ (cited 174 times) further confirmed the regulatory effects of hypoxia and HIF-2α on cartilage differentiation.

NO is a key gas signaling molecule, whereas ROS are oxygen metabolism–related reactive species that participate in cartilage injury and repair. Hashimoto et al.^43^ (cited 307 times) were the first to directly link NO to the apoptosis of chondrocytes and matrix degradation in osteoarthritis. LeGrand et al.^46^ (cited 183 times) found that menisci from patients with osteoarthritis can spontaneously produce NO. Attur and Patel^50^ (cited 158 times) confirmed that IL-17 can enhance NO production through the NF-κB pathway. Pelletier et al.^51^ (cited 149 times) validated in a canine model of osteoarthritis that inhibiting iNOS can broadly reduce inflammation and lessen cartilage damage. Henrotin et al.^42^ (cited 420 times) pointed out that excessive ROS can induce oxidative stress, leading to cell death and matrix degradation, while moderate hypoxia can mitigate its adverse effects. The latest research by Xue et al.^59^ (cited 99 times) utilized ROS-responsive nanomedicine to eliminate ROS, effectively reshaping the inflammatory microenvironment and delaying cartilage damage.

A study by Li et al.^56^ (cited 108 times) demonstrated that H_2_S plays a complex regulatory role in joint inflammation, with its donor capable of inhibiting the production of inflammatory mediators, and the anti-inflammatory effects being influenced by the timing of administration. Related inflammatory factors (e.g., IL-1β, TNF-α, and IL-6) interact with gas molecules. Melchiorri et al.^44^ (cited 215 times) emphasized that chondrocytes are the primary source of these inflammatory mediators in osteoarthritis. Clements et al.^45^ (cited 186 times) found through gene knockout experiments that these factors play a regulatory role in maintaining normal cartilage homeostasis, and solely targeting their inhibition may be counterproductive.

Furthermore, several related studies have provided new directions for cartilage repair. Khan et al.^48^ (cited 174 times) found that a hypoxic environment can optimize the chondrogenic differentiation of stem cells. Chan et al.^52^ (cited 138 times) provided mechanisms for the application of drugs such as glucosamine, confirming that they exert anti-inflammatory effects by reducing the synthesis of NO and PGE_2_. Yan et al.^55^ (cited 113 times) proposed a nanotherapy strategy that reprograms macrophage phenotypes by eliminating NO and inhibiting related enzymes. Pelletier et al.^58^ (cited 101 times) explored the mechanisms of chondrocyte death in osteoarthritis, while Chen et al.^60^ (cited 99 times) reported on the potential of novel biomaterials in cartilage replacement. These studies collectively reveal the significant application value of gas molecules and related factors in the repair of cartilage damage.

**Figure 7:**
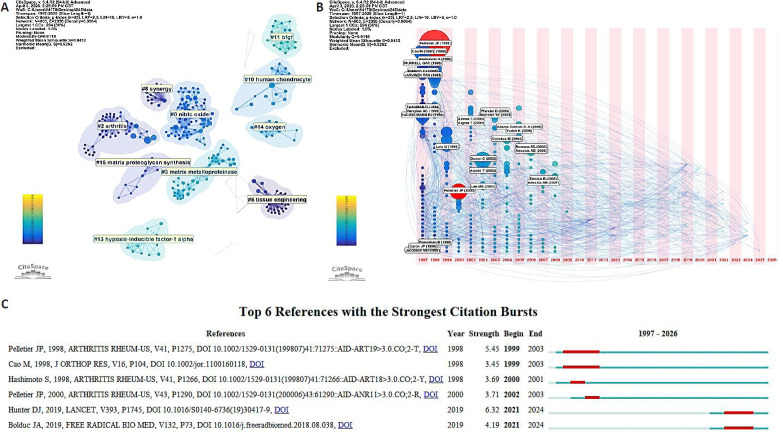
Co-citation visualization analysis of the research on the roles of gas molecules and related factors in knee cartilage injury and repair. (A) Co-citation clustering map: Each node represents a cited paper, with the size of the node positively correlated to its citation frequency. Larger nodes indicate higher citation counts and greater academic influence (indicating highly cited classic literature in the field). The lines connecting two nodes represent a co-citation relationship (i.e., both papers are cited by other works simultaneously). Thicker lines indicate a stronger co-citation intensity, suggesting a closer thematic connection between the two papers. Different colored translucent regions are used to define 10 distinct research clusters. Each cluster is marked with a yellow label to emphasize the core themes that correspond to specific research directions within the field. (B) Panoramic view of co-citation clustering over time: The horizontal axis represents years (1997–2026), with literature nodes arranged vertically according to their first citation year. Larger nodes indicate higher citation counts, signifying highly cited classic literature or milestone achievements in the field. The two large red nodes in the figure are the most cited core papers. Cool colors (blue/cyan) represent early citations, while warm colors (red/yellow) indicate recent citations. These colors also correspond to different clusters (research themes), with red typically representing the core cluster. The year a node appears indicates the first year the paper was cited in the field, establishing the knowledge foundation at that time. Lines represent co-citation relationships between two papers, indicating that they were cited together by a third work. Thicker lines signify a higher frequency of simultaneous citations, indicating a stronger academic connection. The color of the lines corresponds to the year of the first co-citation, with cool colors (blue) indicating early co-citation and warm colors (red) indicating recent co-citation. (C) Burst detection map of co-cited references. “Strength” indicates the intensity of citation bursts (higher values denote more significant bursts and greater impact). “Begin” and “End” mark the start and end years of each citation burst. The timeline on the right (1997–2026) visually represents the burst periods: thick red bars indicate active burst periods, while thin light blue bars indicate non-burst periods.

The results of bibliometric clustering analysis are shown in **Figure 7A**, which reveals a total of 10 clusters: #0 nitric oxide, #2 arthritis, #3 matrix metalloproteinase, #5 tissue engineering, #8 synergy, #10 human chondrocyte, #11 bFGF, #13 hypoxia-inducible factor-1 alpha, #14 oxygen, and #15 matrix proteoglycan synthesis. As shown in **Figure 7A**, the core cluster #0 (nitric oxide) represents the central research line in this field, focusing on the gas molecule NO and its connections to cartilage damage, inflammation, and repair. The other clusters revolve around this central theme, forming several sub-research directions related to pathological mechanisms (arthritis, MMPs, and hypoxia), repair technologies (tissue engineering and growth factors), cellular models (human chondrocytes), and matrix metabolism (proteoglycan synthesis).

There are intersections among different research areas (for example, the relationship between NO and arthritis, and the association between HIF-1α and oxygen concentration). The gas molecules #0 (NO) and #14 (O_2_) are the core gas molecule studies. Related factors #11 (bFGF), #13 (HIF-1α), and #3 (MMPs) are key regulatory factors. Application scenarios #2 (arthritis), #10 (human chondrocytes), and #5 (tissue engineering) represent the clinical/experimental contexts of cartilage damage and repair.

**Figure 7B** presents a panoramic view of the co-citation clustering timeline. The evolution of research clusters is divided into three phases: (1) Foundational period (1997–2005): Core researchers including Pelletier et al.,^51^,^58^ Cao et al.,^62^,^63^ and Hashimoto et al.^43^ focused on verifying the fundamental mechanisms of NO gas molecules related to cartilage damage and repair (regulating apoptosis, inhibiting collagen synthesis, and the iNOS pathway), establishing a core theoretical framework for the field. (2) Development period (2005–2010): The number of publications surged, and connections among them became denser. Research expanded from a single gas molecule (NO) to encompass low oxygen microenvironments^64^ and interactions among multiple gas molecules, leading to a more detailed exploration of the underlying mechanisms. (3) Deepening period (2010 to present): High-quality publications continue to emerge, focusing on oxidative stress,^65^ inflammation regulation, and clinical translation.

**Figure 7C** presents a burst detection map of co-cited references, illustrating the evolutionary trajectory of research hotspots in the field of cartilage injury and repair. Phase 1 (1999–2003): Foundational period. During this phase, scholars represented by Pelletier et al.^66^,^67^ published a series of highly cited and high-burst classic papers in the field of osteoarthritis/rheumatoid arthritis, thereby establishing the core knowledge framework in the field. The study by Pelletier et al.^66^ became the first burst point in the field, marking NO as the core gas molecule in cartilage injury and repair, and launching research in this direction. The study by Cao,^62^ which burst in the same year as that by Pelletier,^66^ verified the regulatory role of NO in cartilage matrix metabolism, further reinforcing the central position of NO. Concurrently, the study by Hashimoto et al.^43^ focused on the association between NO and chondrocyte apoptosis, which completes the core pathway of gas molecule-mediated injury. The study by Pelletier et al.^67^ further clarified how NO activates the caspase-3 apoptosis pathway, providing deeper insight into the underlying mechanisms. Phase 2 (2021–2024): Frontier period. Emerging studies by Hunter et al.^68^ and Bolduc et al.^65^ have become current research hotspots, reflecting the most recent directions in the field. The study by Hunter et al.^68^ has emerged as the strongest burst point in recent years, marking the entry of osteoarthritis research (including the gas molecule direction) into a phase of programmatic summarization, with sustained and growing attention in the field. Studies by Bolduc et al.^65^ and Hunter et al.^68^ burst simultaneously and focus on oxidative stress, gas molecules, and cartilage aging, which are a core frontier hotspot in recent years.

During the intermediate gap period from 2004 to 2020, no high-burst literature emerged, indicating that no milestone achievements capable of leading the field appeared during this phase. Research activity remained relatively stable, without any explosive hotspots.

The top 10 co-cited publications are listed in **Table 7**. All 10 papers were published in leading journals within the field, including the prestigious general medicine journal *The Lancet*, the top rheumatology journal *Arthritis and Rheumatism*, and the leading orthopedic journal *Osteoarthritis and Cartilage*, indicating a high level of authority in their findings. Among the citation data, the most cited individual paper received 3609 citations (Hunter et al., 2019),^68^ and 6 papers were cited over 200 times, with 3 review articles cited more than 500 times. This demonstrates that these papers are foundational and seminal works in the field, widely recognized and cited by the academic community.

**Table 7:** Top 10 co-cited publications addressing the roles of gas molecules and related factors in knee cartilage injury

First author (publication year)	Article title	Source journal	Document type	Co-citation counts	Times cited, WoS Core
Hunter (2019)^68^	Osteoarthritis	*The Lancet*	Review	12	3609
Pelletier (1998)^66^	Reduced progression of experimental osteoarthritis *in vivo* by selective inhibition of inducible nitric oxide synthase	*Arthritis and Rheumatism*	Article	11	309
Bolduc (2019)^65^	Reactive oxygen species, aging and articular cartilage homeostasis	*Free Radical Biology and Medicine*	Review	8	526
Cao (1998)^62^	Generation of nitric oxide by lapine meniscal cells and its effect on matrix metabolism: stimulation of collagen production by arginine	*Journal of Orthopaedic Research*	Article	7	41
Ansari (2020)^69^	Oxidative stress and inflammation in osteoarthritis pathogenesis: role of polyphenols	*Biomedicine Pharmacotherapy*	Review	6	556
Domm (2002)^64^	Redifferentiation of dedifferentiated bovine articular chondrocytes in alginate culture under low oxygen tension	*Osteoarthritis and Cartilage*	Article	6	211
Pelletier (2000)^67^	Selective inhibition of inducible nitric oxide synthase reduces progression of experimental osteoarthritis *in vivo* – possible link with the reduction in chondrocyte apoptosis and caspase 3 level	*Arthritis and Rheumatism*	Article	6	188
Hashimoto (1998)^43^	Chondrocyte apoptosis and nitric oxide production during experimentally induced osteoarthritis	*Arthritis and Rheumatism*	Article	6	307
Cao (1997)^63^	Nitric oxide inhibits the synthesis of type-II collagen without altering Col2A1 mRNA abundance: prolyl hydroxylase as a possible target	*Biochemical Journal*	Article	6	143
Blanco (1998)^70^	Osteoarthritis chondrocytes die by apoptosis: a possible pathway for osteoarthritis pathology	*Arthritis and Rheumatism*	Article	5	574

Co-citation counts refer to the number of times two articles are cited together by a third article. A higher value indicates greater academic impact and significance of the cited articles in the field, marking them as foundational literature. "Times cited, WoS Core" represents the total citation counts of an article within the Web of Science Core Collection, reflecting the global academic impact of the publication.

All publications in **Table 7** focus on the core pathology of cartilage degeneration and injury repair, addressing NO as a core gas signaling molecule and ROS as oxygen metabolism–related reactive species, together with their upstream and downstream regulatory factors. Five major research hotspots are summarized in **Box 1**. As shown in **Box 1**, NO induces chondrocyte apoptosis through the iNOS–NO–caspase3 axis (hot topics 1, 2, and 4), while oxidative stress driven by ROS further exacerbates this process (hot topic 3). These gas signaling and oxygen metabolism–related factors ultimately exert direct effects on matrix metabolism (hot topic 5), collectively determining the outcomes of cartilage damage and repair.

**Box 1: Core research trends and hot topics in gas signaling molecules and related regulatory factors**

**Hot topic 1: Nitric oxide (NO) is a key gas molecule involved in mediating cartilage damage**

NO can directly inhibit the synthesis of type II collagen, disrupt cartilage matrix metabolism, and induce apoptosis in chondrocytes, thereby promoting the progression of osteoarthritis. Key literature by Cao et al.,^62^,^63^ Hashimoto et al.,^43^ and Blanco et al.^70^ has established the central role of NO in cartilage injury.

**Hot topic 2: Inducible nitric oxide synthase–nitric oxide (iNOS–NO) axis**

iNOS is a critical enzyme responsible for the pathological production of NO. Selective inhibition of iNOS can significantly slow the progression of experimental osteoarthritis, reduce chondrocyte apoptosis, and lower caspase-3 levels. This is primarily supported by two *in vivo* studies conducted by Pelletier et al.^66^,^67^

**Hot topic 3: Reactive oxygen species (ROS) and oxidative stress are important driving factors in age- and inflammation-related cartilage degeneration**

Excessive generation of ROS disrupts cartilage homeostasis, with oxidative stress and inflammation mutually exacerbating cartilage damage. Antioxidants, such as polyphenols, have potential therapeutic potential for cartilage damage. Similar conclusions are drawn from two reviews by Bolduc et al.^65^ and Ansari et al.,^69^ while Domm et al.^64^ demonstrated that a hypoxic environment can promote chondrocyte redifferentiation.

**Hot topic 4: Chondrocyte apoptosis is a core downstream pathway through which gas molecules damage cartilage**

Chondrocyte apoptosis is an important pathological feature of osteoarthritis, with NO serving as a key upstream factor inducing apoptosis. The iNOS–NO–caspase 3 pathway is the central regulatory mechanism involved. Publications supporting this conclusion include studies by Blanco et al.,^70^ Hashimoto et al.,^43^ and Pelletier et al.,^67^ while Hunter et al.^68^ further confirms this as a consensus in the field.

**Hot topic 5: Gas molecules directly regulate cartilage matrix metabolism, determining the outcomes of damage and repair**

NO inhibits collagen synthesis and disrupts matrix balance, whereas an appropriate hypoxic microenvironment promotes chondrocyte redifferentiation and restores matrix synthesis capacity. Targeted modulation of NO and ROS can reverse the imbalance of matrix degradation. Key literature includes studies by Cao et al.,^62^,^63^ Domm et al.,^64^ and Pelletier et al.,^66^,^67^ along with relevant reviews.

## Discussion

### Result analysis and significance

In the past 30 years, the volume of publications of research on gas molecules and related factors in knee cartilage injury and repair has shown a steady growth trend, with China becoming a core contributor to research output. However, its centrality remains low at 0.08, indicating that its role as a hub for international collaboration and academic discourse is still significantly weaker than that of the United States and the United Kingdom. The latter, benefiting from an early start and high centrality, occupy a pivotal position in the international cooperation network. Institutes such as the Scripps Research Institute in the United States and Sichuan University in China lead the field with six publications each. However, high-output institutions in China generally face the issue of low impact despite a high number of publications; for instance, Sichuan University has six papers with a total citation count of less than 30, which is significantly lower than that of similar institutions such as Scripps Research Institute. By contrast, institutions such as Seoul National University in South Korea and the University of Manchester in the United Kingdom, despite having fewer publications, have achieved high-impact results, establishing themselves as academic benchmarks. Moreover, high-citation institutions tend to focus on independent research, with very low levels of inter-institutional collaboration, reflecting a mismatch between research output and impact, as well as significant collaboration barriers within the field. Journal analysis indicates that *Osteoarthritis and Cartilage* is the core journal in this area, with its research focus shifting from traditional pathological mechanisms to translational applications in biomaterials and nanomedicine. In recent years, interdisciplinary integration has become a core trend in journal publication direction.

The keyword co-occurrence analysis in this study shows a high degree of consistency with both the highly cited literature and the literature co-citation clustering results, forming a complete research logic loop across three dimensions: core themes, pathological mechanisms, and translational directions. (1) At the dimension of core themes: Keywords such as “osteoarthritis,” “chondrocyte,” and “cartilage” rank at the top of the list with the highest frequencies. These keywords correspond closely with the pathological core of cartilage injury in osteoarthritis highlighted by highly cited literature, collectively establishing a research framework focusing on “disease–target cell–target tissue.” (2) At the dimension of pathological mechanisms: High-frequency keywords including “nitric oxide,” “reactive oxygen species,” “hypoxia,” “oxidative stress,” and “apoptosis” correspond one-to-one with the five core research hotspots identified in the highly cited literature. They also fully match the key clusters identified in literature co-citation analysis, such as Cluster #0 (nitric oxide), Cluster #14 (oxygen), and Cluster #13 (hypoxia-inducible factor-1α). These findings jointly reveal the core scientific issues of the field: NO, through the iNOS-NO-caspase 3 pathway, induces chondrocyte apoptosis and inhibits type II collagen synthesis, establishing itself as the core gas molecule mediating cartilage injury. Excessive ROS trigger oxidative stress and inflammatory responses that synergistically exacerbate cartilage degeneration, serving as a key driver of age-related osteoarthritis. The hypoxic microenvironment, mediated by hypoxia-inducible factors, regulates chondrocyte differentiation and homeostasis, exerting both detrimental and protective effects. Notably, although H_2_S shares similar gas-signaling properties with NO and holds potential for anti-inflammatory and anti-apoptotic effects in cartilage protection. Bibliometric analysis reveals that its publication volume remains far lower than that of NO, and it has not yet formed a highly cited cluster. This suggests that research on H_2_S in this context is still in its early stages. Although O_2_ appears with relatively low frequency as a keyword, its intrinsic relationship with hypoxia and the HIF pathway is reflected in Cluster #14 (oxygen), collectively constituting an oxygen metabolism regulatory network. (3) At the dimension of translational directions: Emerging hot keywords such as “graphene oxide,” “cartilage repair,” and “tissue engineering” are highly consistent with frontier research directions identified in the highly cited literature, including nanomedicine, novel hydrogel materials, and stem cell–based cartilage differentiation. These terms also correspond well with key clusters such as Cluster #0 (cartilage tissue engineering), Cluster #2 (hyaluronic acid), and Cluster #5 (chitosan scaffold). Together, they highlight a clear evolutionary trend in the field: shifting from basic mechanistic studies toward clinical translation, and from single-target strategies to multidisciplinary approaches. In particular, the integration of gas-signaling molecule regulation with biomaterials and regenerative medicine has become one of the most promising frontiers in this research area.

It is worth noting that ozone therapy has been identified as a hot topic in keyword analysis; however, no highly cited literature related to this topic was detected in the analysis of literature hotspots. The specific reasons are as follows: keyword analysis focuses on the frequency of theme occurrences across all literature without a citation threshold, reflecting the breadth of attention in the academic community.^71^,^72^,^73^ According to the results, ozone therapy has emerged as a new direction since 2016 and has been frequently mentioned in several papers. In contrast, the literature hotspot analysis focuses exclusively on highly cited milestone achievements, most of which were published during 1997–2013. Research on ozone therapy, however, has only emerged more recently. As a result, these newer publications have not yet had sufficient time to accumulate enough citations to be captured as major hotspots. Additionally, much of this work consists of application verification studies within existing theoretical frameworks and has not produced foundational theoretical breakthroughs. As a result, individual citations have not reached the selection threshold, leading to the absence of highly cited literature in this area. Thus, ozone therapy for cartilage damage in the knee is considered an “emerging theme” rather than a “classic hotspot” or “knowledge foundation.” It has not yet produced any milestone foundational results, indicating a slight lack of theoretical depth.^74^,^75^

### Study limitations

The literature data is sourced solely from the Web of Science Core Collection database and does not include documents from other Chinese and English databases such as CNKI and Scopus. It also does not cover gray literature such as theses, conference abstracts, or patents, which limits the scope of the literature and may introduce some bias in analyzing clinical research and technology transfer outcomes in China. Given that this study aims to reveal research hotspots and knowledge structures in the international academic community, the Web of Science Core Collection database is widely regarded as a mainstream platform for monitoring cutting-edge trends in the fields of natural sciences and clinical medicine, lending a degree of reliability to the literature results. Our bibliometric data support the existence of co-occurrence patterns, but causal interaction networks require experimental validation.

Additionally, the latest literature included from 2023 to 2026 has not yet accumulated sufficient citation data due to its short publication period, which may lead to a lag in assessing the impact of emerging frontier hotspots. The article adopts a relatively conservative judgment principle when interpreting emerging trends (such as nanomedicine) to avoid premature conclusions about short-term fluctuations or immature directions. In rapidly evolving fields such as nanomedicine, low citation counts do not equate to low value; rather, they indicate the need for further tracking using multidimensional data, including funding, preprints, and patents. The article emphasizes that these areas are still in their nascent stages and that impact assessments must be dynamically updated. Moreover, emerging gas molecules such as H_2_S have not significantly appeared in clustering due to the current limited volume of publications. This is not an oversight but a result of the objective requirements of clustering algorithms regarding sample size. Such themes currently lack the stability and density needed to form a robust co-citation network. Future research will conduct specialized analyses once sufficient literature has accumulated. In summary, the article aims to reliably identify core hotspots that have reached a certain scale and can be captured by mature citation networks within the domain of international high-quality journal literature. For emerging, interdisciplinary, or localized issues, the article maintains an open attitude and explicitly points out the necessity of expanding data sources and extending the observation window in future work. This approach aligns with the conventional norms of bibliometric research and does not affect the reliability and reference value of the conclusions of the article within its defined scope.

It is noteworthy that although H_2_ and H_2_S were mentioned in the Introduction as emerging gasotransmitters with chondroprotective potential, supported by recent mechanistic studies demonstrating H_2_-mediated mitochondrial restoration and H_2_S-induced MMP downregulation—these molecules did not appear as distinct clusters or high-frequency keywords in our bibliometric results. This absence is not an oversight but rather an objective reflection of the current publication landscape: research on H_2_ and H_2_S in knee cartilage injury and repair is still in its nascent stage, with insufficient cumulative publication volume and citation density to reach the thresholds required for co-citation network formation or burst detection. This quantitative gap paradoxically underscores their promise as a “next frontier” in the field. As the number of studies on these gases continues to grow, we anticipate that they will gradually form identifiable clusters in future bibliometric analyses, potentially revealing novel mechanisms—such as Nrf2-mediated antioxidant pathways and mitochondrial quality control—that may complement or even redefine the current NO–ROS–centered paradigm. We therefore encourage researchers to accelerate investigations into H_2_ and H_2_S, particularly through interdisciplinary collaborations integrating gas biology with biomaterials and targeted delivery systems, to transform these emerging candidates into clinically translatable strategies for cartilage repair. Furthermore, our analysis reveals that most studies concentrate on osteoarthritis models, with limited attention to acute traumatic injury. This imbalance suggests an opportunity for future investigations into gas molecule regulation in the early phase of cartilage trauma.

### Conclusions and prospects

This article uses bibliometric and visualization analysis methods to outline the development trends in the research field of gas molecules and related factors in osteoarthritis from 1997 to 2026. The core research in this field focuses on the mechanisms of cartilage injury repair mediated by NO, ROS, and hypoxia. Future studies may benefit from analyzing the multi-factor interaction network formed by NO, H_2_S, ROS, HIF, inflammatory factors, and apoptotic pathways, rather than studying individual gas molecules in isolation.

The research focus has shifted from “how gas molecules damage cartilage” to “how to use gas molecules (and materials that regulate their levels) to reshape the damaged microenvironment.” Researchers need to further deepen the investigation into the core mechanisms of multi-factor interaction networks, strengthen international collaboration, and leverage institutional advantages to overcome technical bottlenecks in targeted delivery and biomaterials. This will facilitate the translation of gas-targeted regulatory strategies into clinical diagnosis and treatment for osteoarthritis, providing new solutions for cartilage injury repair.

## Data Availability

*Not applicable.*
